# Toward sustainable phycocyanin production using halo-alkaliphilic cyanobacteria: from direct air capture of carbon dioxide to biorefinery

**DOI:** 10.3389/fmicb.2025.1618123

**Published:** 2025-07-23

**Authors:** Lianchun Yi, Ruchita Solanki, Miranda Moll, Agasteswar Vadlamani, Hector De la Hoz Siegler, Marc Strous

**Affiliations:** ^1^Department of Earth, Energy, and Environment, University of Calgary, Calgary, AB, Canada; ^2^Department of Chemical and Petroleum Engineering, Schulich School of Engineering, University of Calgary, Calgary, AB, Canada

**Keywords:** phycocyanin, alkaline soda lake, cyanobacteria, direct air capture, biorefinery

## Abstract

Phycocyanin is a natural blue pigment from cyanobacteria such as *Limnospira platensis*, also known as “Spirulina.” Its production is costly and faces sustainability challenges due to water needs, carbon dioxide emissions, and lack of operational stability. Here, we review the use of halo-alkaliphilic cyanobacteria to overcome these challenges. This review synthesizes conceptual innovations that were proposed and tested experimentally previously, resulting in the presentation of a complete bioprocess for phycocyanin production. These innovations are: (1) the use of a pH above 10.5 to implement direct air capture of carbon dioxide, reducing carbon dioxide emissions; (2) the use of a consortium of an alkaliphilic cyanobacterium and its associated heterotrophs for improved process stability; (3) the use of passive fermentation for phycocyanin extraction, thereby reducing water needs; and (4) the use of anaerobic digestion to recover energy and recycle carbon dioxide and nutrients. Integrating the above approaches could offer a potentially scalable, more sustainable alternative to conventional phycocyanin production, aligning with circular bioeconomy goals. Several challenges still require solutions. For example, despite water savings, water losses associated with direct air capture of carbon dioxide remain high, and nutrient recycling is only partially successful so far.

## Introduction

Phycocyanin is a natural blue protein pigment that is used as a food colorant. With growing consumer demand for natural ingredients, phycocyanin is often marketed as a healthy, proteinaceous alternative to synthetic dyes. Despite its sensitivity to heat and acidity (Yuan et al., 2022), it is attractive due to its antioxidant potential and vibrant color.

In practice, the main commercial source of phycocyanin is *Limnospira platensis*, a filamentous, photosynthetic cyanobacterium formerly known as *Arthrospira platensis* and commonly referred to as “Spirulina.” *L. platensis* is valued not only for its phycocyanin content but also for its high protein content (50–70%) (Williams and Phillips, 2014; Lupatini et al., 2017). Historically, its dried biomass was consumed by Indigenous Nations surrounding saline or soda lakes such as Lake Texcoco in Mexico and Lake Chad in Central-Western Africa (Ciferri, 1983). It is designated “generally recognized as safe” by the U. S. Food and Drug Administration (FDA) (FDA, U. S., 2024).

Despite its commercial success, the large-scale production of phycocyanin remains both environmentally and economically challenging. To produce 1 kg of *L. platensis* biomass requires 800 liters of water (Girotto et al., 2021). As cyanobacterial biomass contains approximately 10–20% phycocyanin (Chaneva et al., 2007), the water consumption per kilogram of phycocyanin powder is even higher. Cultivating *L. platensis* also requires continuous sparging with external carbon dioxide, e.g., flue gas and energy-intensive drying for biomass dewatering. The latter step requires substantial energy inputs, driving up production costs. As a result, the production of phycocyanin costs as much as $250 U. S. dollars per kilogram (Chaiklahan et al., 2018). This, together with its instability at low pH and high temperature, ultimately limits the broader adoption of phycocyanin pigment.

Given the increasing interest in the biotechnology of alkaline environments, we review the potential of alkaliphilic cyanobacteria to improve the sustainability of phycocyanin production. We identify several potential conceptual advantages that have been explored experimentally in laboratory settings; some have also been explored at the technical scale. It is not yet possible to quantitatively estimate the potential benefits using techno/economic feasibility analysis or life cycle assessment because: (1) this is still a very early-stage biotechnology; and (2) to date, the literature lacks reference case data of commercial-scale phycocyanin production. The use of alkaliphilic cyanobacteria is by no means the only way to improve the sustainability of phycocyanin production. For example, synthetic biology offers interesting approaches as well (e.g., Hudson, 2021; Markley et al., 2015), but these are not the focus of this review.

**Figure 1:**
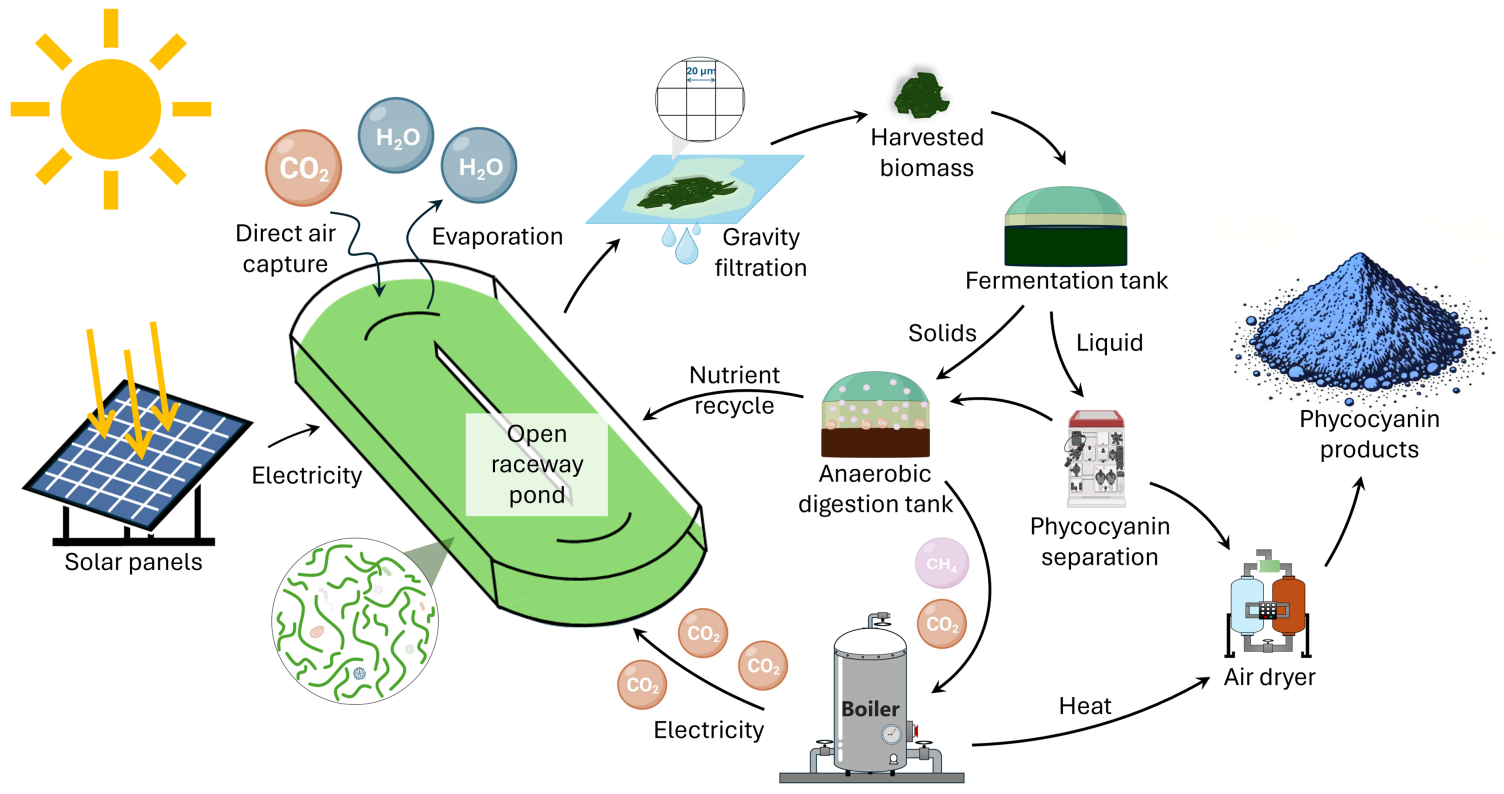
A proposed bioprocess for producing phycocyanin from alkaliphilic cyanobacteria.

*L. platensis* originates from an alkaline soda lake and is typically grown at a pH of 9.5. Since 2013, several labs have explored the benefits of producing phycocyanin from different cyanobacterial species that grow at an even higher pH (see Canon-Rubio et al., 2016 and references therein). Integrating all this research, a potential bioprocess emerges as illustrated in Figure 1. The cyanobacteria grow in a raceway pond at a pH of 10.5 or higher. The pond captures carbon dioxide for growth directly from the air. The biomass harvest happens continuously using a screen overlying the pond, with spent medium percolating back into the pond. The harvested biomass ferments spontaneously, leading to the rapid lysis of the cyanobacterial cells and the passive release of the phycocyanin, which is separated and purified via filtration. During the fermentation, the pH decreases below 8, which enables anaerobic digestion of the solids and liquid remaining after phycocyanin separation. A combined heat and power unit combusts the biogas, producing heat for phycocyanin drying and contributing electricity for the operations. The produced carbon dioxide is recycled into the pond, supplementing air capture. As is the digestate, which enables the recycling of most of the nutrients. This bioprocess potentially outperforms conventional phycocyanin production, because of increased operational stability, reduced carbon dioxide emissions, and reduced water and nutrient needs.

## Bioprospecting cyanobacteria from alkaline soda lakes

**Figure 2:**
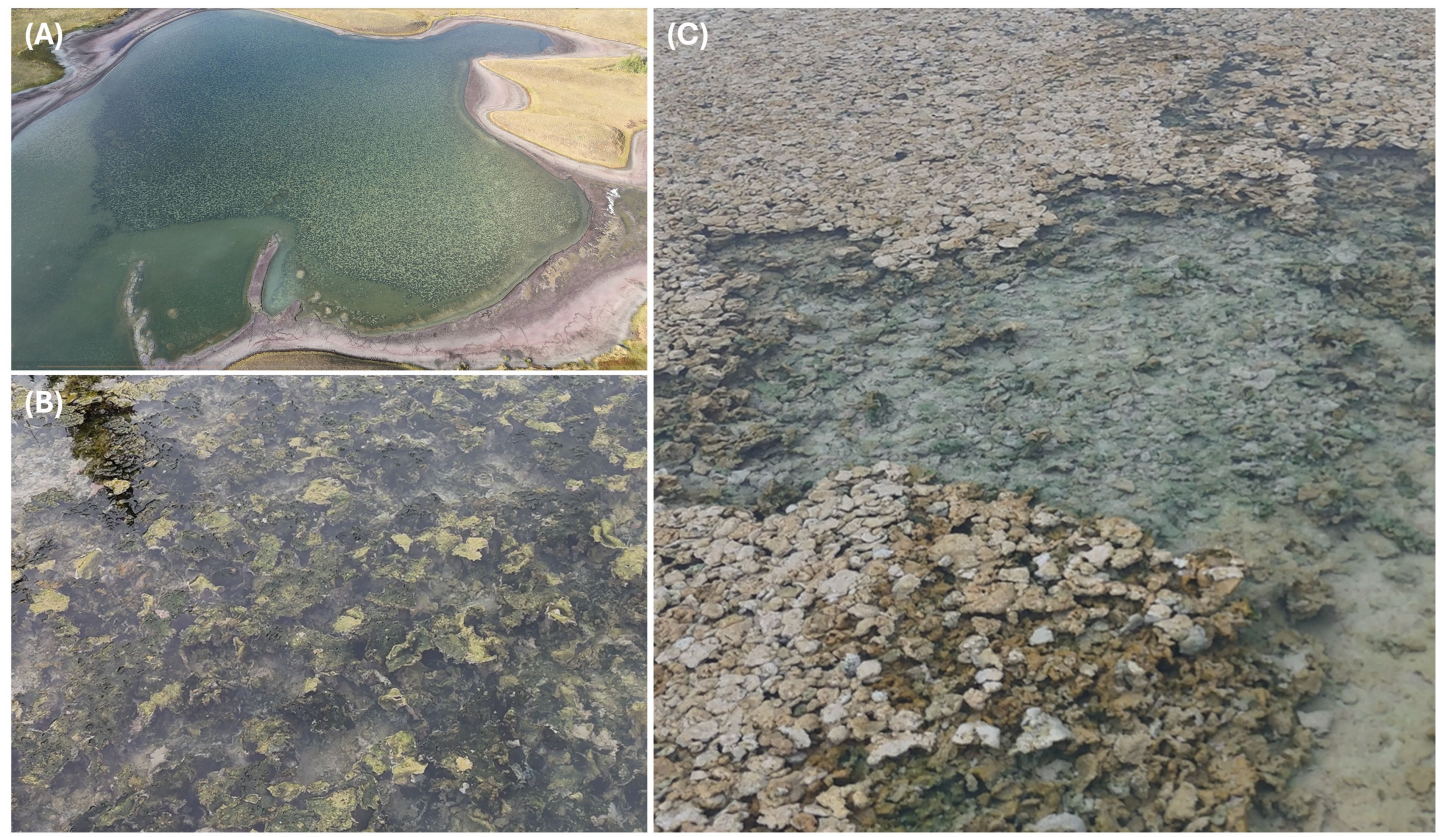
Microbial mats in alkaline soda lakes in British Columbia, Canada. **(A)** Goodenough Lake; **(B)** Probe Lake; and **(C)** Deer Lake.

Alkaline soda lakes are the main natural habitat of high-pH-adapted cyanobacteria. These lakes are high-pH environments, where cyanobacteria microbial mats can form at the sediment–water interface. Figure 2 shows the microbial mats from three of many alkaline soda lakes in the Cariboo Plateau region of Canada (Brady et al., 2013; Zorz et al., 2019). Soda Lakes also exist in many other places, such as Central Asia, Africa, and South America. One of the earliest isolated alkaliphilic cyanobacteria, *Euhalothece* sp. Z-M001, originated from Lake Magadi in Eastern Africa (Mikhodyuk et al., 2008). This cyanobacterium was the first to be grown in a photobioreactor with integrated carbon dioxide capture (Kishi and Toda, 2018). Another promising high-pH-adapted genus of cyanobacteria is Sodalinema (Koch et al., 2022; Nies et al., 2022; Samylina et al., 2014; Samylina et al., 2021). Representatives were isolated from both alkaline soda lakes and marine environments. Finally, a coccoid, unicellular alkaliphilic species of *Cyanobacterium* was isolated for use in high-pH biotechnology (Gao et al., 2023). All these bacteria grow above pH 10.5 and could be used to drive direct air capture of carbon dioxide in photobioreactors or open raceway ponds. Two relevant criteria for bioprospecting cyanobacteria from alkaline soda lakes are the growth rate and the cell morphology. With regard to growth rate it is important to consider the pH optimum of growth, the tolerance to light, alkalinity, and sensitivity to other environmental conditions. With regard to morphology, harvesting and dewatering will be much more effective for large filamentous cyanobacteria such as *Limnospira* and *Sodalinema*, than for smaller coccoid species. Species might also differ in the amount and color of the phycocyanin that can be extracted. A systematic comparison of alkaliphilic cyanobacteria regarding these criteria has not yet been completed.

## Stability benefits of using alkaliphilic isolates and consortia

Industrial cultivation of cyanobacterial isolates frequently deals with the challenge of culture invasion. This is largely due to the fact that to reach large-scale phycocyanin production, cyanobacterial cultivation eventually takes place in large, open raceway ponds. Growth in such open-air systems will inevitably lead to the reintroduction of heterotrophs from the local environment. These invading heterotrophs can cause rapid culture crashes (Troschl et al., 2017) and may become an unregulated component of the phycocyanin product. This compromises both system robustness (Shurin et al., 2013) and product safety. Prevention of heterotroph or pathogen invasion requires intentional design and operational practices, with detection and identification of contaminants adding to costs (Molina-Grima et al., 2022). In dealing with an invasion, many treatment options, such as chemical control agents and pesticides (Molina-Grima et al., 2022), compromise both the product’s quality and the sustainability of its production.

Most invading bacteria or protists are adapted to near-neutral pH and lower sodium concentrations (Krulwich et al., 2011; Foissner, 2006). Therefore, alkaline cultivation systems operated at a pH of 10.5 or higher may be more robust to invasion. The high-pH, high-alkalinity environment may effectively filter out non-adapted microorganisms, limiting the success of potential invaders and contributing to process stability and product safety.

There is also debate regarding the use of an isolated cyanobacterium in monoculture versus the use of a cyanobacterial consortium, a consortium of a single cyanobacterium with its natural heterotrophic partners (Mollo et al., 2024). After all, biodiversity is known to improve the stability of a microbial community (Cardinale et al., 2013). In natural ecosystems, such as the microbial mats shown in Figure 2, cyanobacteria support heterotrophic bacteria by releasing organic compounds during growth and after cell death. These heterotrophs, in turn, recycle nutrients that benefit cyanobacteria. Cyanobacteria can form more or less stable consortia with heterotrophic bacteria (Paerl et al., 2000). When attempting to grow a cyanobacterium in monoculture at large scale, invasion of heterotrophic bacteria is unavoidable. However, relationships between a cyanobacterium and opportunistic invading heterotrophic species may be less beneficial, or even harmful, compared to when a cyanobacterium grows together with heterotrophic bacteria as a natural consortium, with partnerships that have evolved over millions of years.

**Figure 3:**
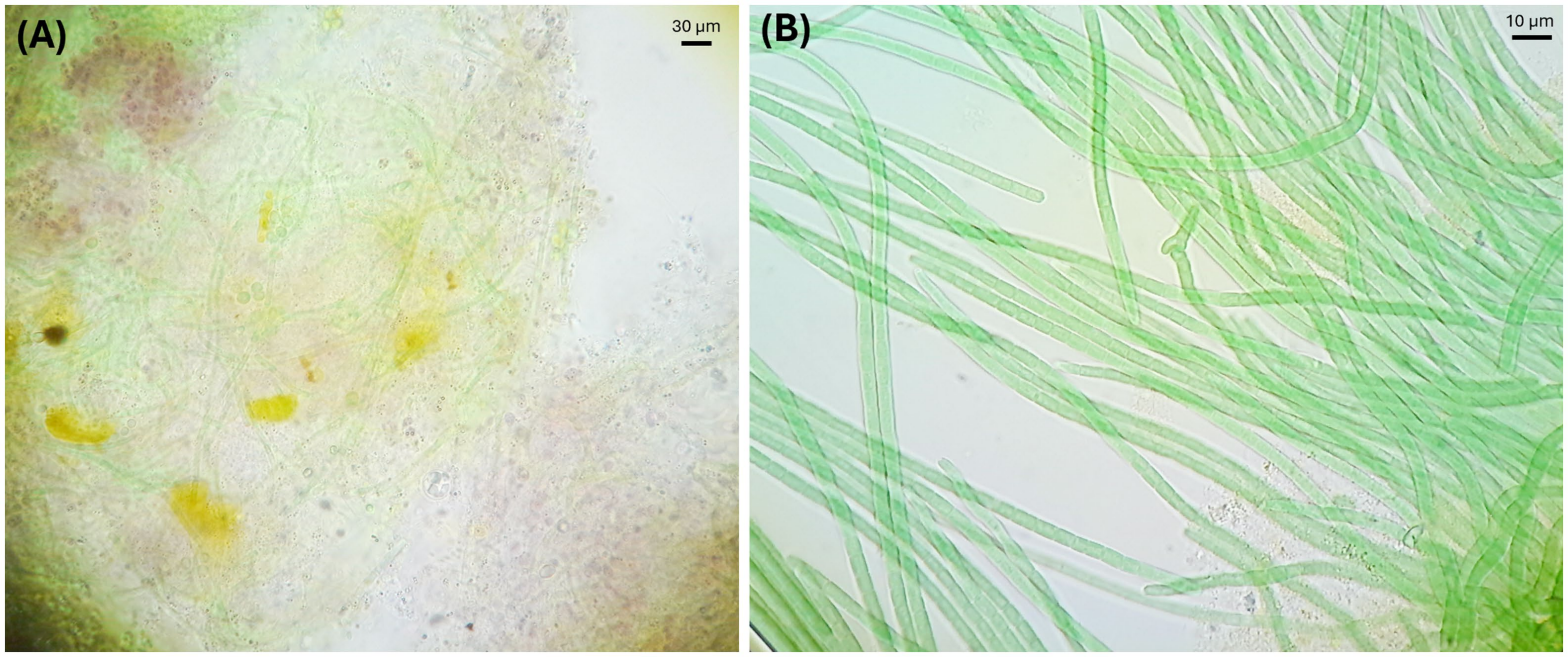
Alkaline microbial community microscopic images. **(A)** Microbial community in soda lake microbial mats. **(B)** Enriched microbial community from soda lake microbial mats.

The ecological robustness of an alkaliphilic cyanobacterial consortium growing at high pH has been addressed experimentally. A consortium of “*Candidatus Sodalinema* alkaliphilum” and its natural heterotrophic partners was enriched from Canadian soda lakes (Figure 3). It was cultured in planar, tubular, and stirred photobioreactors (PBRs) using an alkaline growth medium with pH > 10 (Ataeian et al., 2019; Ataeian et al., 2022; Ataeian et al., 2021; Sharp et al., 2017). The heterotrophic partners included the genera *Wenzhouxiangella* (Sorokin et al., 2020) and *Cyclonatronum* (Zhilina et al., 2023). No culture crashes resulting from invasion were observed during three seasons of outdoor growth of the *Sodalinema* consortium in raceway ponds and photobioreactors (Haines et al., 2024; Haines et al., 2022; Strous et al., 2025). The heterotrophs may have contributed to the observed process stability via niche occupation and ecological redundancy (Ataeian et al., 2022). That means that by occupying all available ecological niches, the heterotrophs prevent successful invasion by other, potentially harmful heterotrophic species.

Although productivity losses resulting from invading heterotrophs in large open raceway ponds of *L. platensis* are unknown, one may still argue that the use of a consortium could reduce phycocyanin yield even more. After all, heterotrophs depend on the transfer of carbon from cyanobacteria. In practice, the yield loss was quite small. In the *Sodalinema* consortium, the heterotrophs make up 2–15% of the total biomass, even when grown in an outdoor raceway pond or photobioreactor (Haines et al., 2024; Haines et al., 2022; Strous et al., 2025). This indicates only a small fraction of the carbon is transferred to heterotrophs, a relatively small price for avoiding culture crashes because of invading species. Runaway temperature and pH did still result in a crash in an open raceway pond (Strous et al., 2025), showing that ecological solutions also have their limitations.

Unfortunately, current regulatory requirements still strongly favor the use of an isolated species of cyanobacteria over the use of a consortium.

## Direct air capture of carbon dioxide

Bicarbonate is the main carbon source of many cyanobacteria, including *Limnospira*. With their “carbon-concentrating mechanism,” these cyanobacteria import the bicarbonate across their cell membrane into carboxysomes, where they convert it to carbon dioxide for assimilation (Badger and Price, 2003). The uptake of bicarbonate from the medium reduces its concentration, and the associated release of hydroxide ions (OH^−^) increases the medium’s pH. This is the reason why commercial phycocyanin production facilities control the pH and replenish bicarbonate by sparging concentrated carbon dioxide (flue gas) into their open raceway ponds (White and Ryan, 2015). The flue gas is produced on-site by burning natural gas. Bubbling this flue gas into the pond is associated with carbon dioxide emissions. These emissions could be avoided if carbon dioxide would instead be captured directly from the air.

Earth’s geological record shows air capture of carbon dioxide into alkaline solutions has occurred at massive scales (Tutolo and Tosca, 2018). At pH > 10, direct air capture is driven by the difference between a solution’s actual carbon dioxide activity, determined by pH and carbonate alkalinity, and the equilibrium carbon dioxide activity in the medium, determined by the atmospheric partial pressure. For sufficiently diluted systems, such as fresh water, the activity of carbon dioxide in the liquid phase can be approximated by its molar concentration. However, for concentrated carbonate solutions, the behavior of the aqueous phase is far from ideal, and a Pitzer-style activity model (Toner and Catling, 2020) is required to calculate the driving force for direct air capture. Dissociation constants (pK) determined for dilute natural waters do not describe the non-ideal behavior of concentrated carbonate solutions very well.

To maximize the carbon dioxide capture rate, maintaining a high pH is critical, while sufficient total carbonate alkalinity is required to provide sufficient buffering capacity to maintain air capture rates during the night. At night, cyanobacterial growth stalls and is unable to prevent the decrease in pH caused by carbon dioxide capture. Optimum pH and carbonate alkalinity levels vary by species. For example, *L. platensis* exhibits maximal growth between 8.5 and 9.0 (Hu, 2004), with significant growth inhibition above 9.0 (Ismaiel et al., 2016). This is not compatible with direct air capture. Therefore, the selection of appropriate cyanobacterial species with tolerance to higher pH is imperative.

The *Sodalinema* consortium can balance growth and air capture in an open raceway pond around pH 10.7, at a total carbonate alkalinity between 0.35 and 0.5 mol/L (Strous et al., 2025). Under these conditions, the carbon dioxide capture rate was 1.8 g-C/m^2^/day. For comparison, in an open raceway pond in New Mexico, *L. platensis* can assimilate carbon dioxide at a rate of ~7 g-C/m^2^/day (White and Ryan, 2015). Increasing the pH above 10.7 will increase capture rates but simultaneously reduce the cyanobacterial growth rate. For the *Sodalinema* consortium, it grows up to pH 11.3. At this pH, at a carbonate alkalinity of 0.5 mol/L, the bicarbonate concentration is about 7 mmol/L. Beyond that pH, bicarbonate becomes growth-limiting, leading to extreme light sensitivity and DNA damage (Yi et al., 2024). This suggests that additional optimization may improve outcomes. Ponds versus Photobioreactors.

The industrial production of cyanobacteria is not limited to ponds, photobioreactors are sometimes also used. The *Ca. S. alkaliphilum* consortium has been successfully cultivated in both an outdoor tubular photobioreactor and an outdoor open raceway pond (Haines et al., 2024; Haines et al., 2022; Strous et al., 2025). Currently, almost all phycocyanin is produced in open raceway ponds. It is recognized that open raceway ponds are more cost-effective, even though the efficiency of converting sunlight into biomass is quite low, in the order of 1% in practice. Several factors explain this low efficiency (Ooms et al., 2016). For example, the optimal light intensity for cyanobacteria growth ranges between 100 and 200 W/m^2^. However, incident sunlight at midday can reach intensities around 1,000 W/m^2^. Optimal cell density is critical to prevent photobleaching, which induces cell death. However, excessive cell density leads to light attenuation, resulting in suboptimal illumination for deeper cell layers and consequent biomass lost through dark respiration. This challenge is compounded by diurnal and weather-dependent fluctuations in solar irradiance. Additional factors limiting photosynthetic efficiency include: the narrow spectral range of photosynthetically active radiation, geographical latitude, surface light reflectivity, and bioreactor geometry (Ooms et al., 2016). Indoors, artificially illuminated photobioreactors can be much more efficient, but the capital and energy costs of such systems (Richardson et al., 2014) have so far been too high to enable commercial phycocyanin production. With future innovations, photobioreactor designs may become less costly.

Synergies of using high-pH growth media and direct carbon capture mainly apply to open raceway ponds. First, ponds have a large liquid surface area exposed to the air, which means direct air capture happens spontaneously. For a closed photobioreactor, a dedicated air capture unit (Wang et al., 2024) would need to be integrated, adding to already high capital costs. Second, the large liquid surface area of ponds makes them more vulnerable to invasion. Here, the high pH of the medium reduces that vulnerability by making it more difficult for predators and pathogens to invade, as discussed in the previous section.

Water losses are a key remaining challenge of growing cyanobacteria in open raceway ponds (Kumar et al., 2015). At the Sapphire Energy facility in the midwestern US, the annual evaporation rate is 6 mm/day (White and Ryan, 2015), and it is modeled to be 5 mm/day in southwestern US desert regions (Quiroz et al., 2021). At higher latitudes evaporation rates can be high as well; in Calgary, for example, evaporation rates are 4–6 mm/day (Strous et al., 2025). The water losses amount to ~ 800 L of fresh water per kg of biomass produced (Girotto et al., 2021), which translates to at least 3,200 L per kg of phycocyanin. Direct air capture of carbon dioxide and water evaporation both scale with surface area and mass transfer coefficients, so the uncoupling of water evaporation and air capture is a major remaining challenge.

## Biomass dewatering and extraction of phycocyanin

Downstream processing of cyanobacterial biomass starts with harvesting and dewatering. Currently, gravity filtration is used to harvest and dewater *L. platensis* for phycocyanin production (Yu et al., 2021; Zheng and Zheng, 2015). In this process, the culture is pumped onto a screen, mesh, or filter, with the cyanobacteria being retained and liquid media passing through. Filtration was shown to work well with the *Ca. S. alkaliphilum* consortium grown in an outdoor raceway pond (Strous et al., 2025). Other approaches for dewatering are centrifugation (Prihantini et al., 2018), flocculation (Alam et al., 2016), and electro-coagulation (An et al., 2019), but all of these are more costly and require more energy (Molina-Grima et al., 2003).

In current industrial phycocyanin production, the dewatered biomass is washed and dried. Drying is necessary to transport the biomass from the cultivation facility to the phycocyanin extraction facility. After that, the biomass is rewetted and the cells are mechanically lysed. The phycocyanin is soluble and is separated from cell debris by filtration. Finally, the phycocyanin solution is spray-dried, yielding a blue powder. The two drying steps and mechanical lysis contribute to the high water and energy use and increase costs.

The alkaline and high pH growth media enable a more efficient approach for phycocyanin extraction: Under these extreme conditions, bacteria have higher energy needs to maintain the ion gradients over their cell membrane. When cyanobacteria are then placed in the dark, they can no longer conserve energy from light and are dependent on stored energy (e.g., glycogen) to maintain these ion gradients (Carrieri et al., 2010). For *Ca. S. alkaliphilum,* the energy stores last for about a week; once they are depleted, the cells initiate a genetic program for self-destruction, releasing phycocyanin into the medium (Zorz et al., 2023). Thus, harvested biomass can be incubated statically in the dark for passive phycocyanin extraction (Demirkaya et al., 2023; Vadlamani et al., 2021). During this incubation, *Ca. S. alkaliphilum* performs mixed acid fermentation, converting intracellular storage materials into acetate, propionate, succinate, and lactate. If the initial biomass concentration is high enough, the released acids are able to overcome the carbonate buffer and reduce the pH of the medium to ~7. This is important as it enables the closing of recycling streams later on by anaerobic digestion (Demirkaya et al., 2023). The released phycocyanin can be separated from remaining solids and metabolites by filtration. The same approach also works for *L. platensis* (Vadlamani et al., 2021).

Together, gravity filtration and passive incubation require minimal energy inputs and avoid the need for washing biomass and mechanical lysis. The largest remaining energy expense is associated with spray-drying the phycocyanin solution, the final step of phycocyanin production. Heat and electricity for powering this step can be derived from the biogas produced during anaerobic digestion of what remains of the biomass and metabolites after filtering out the phycocyanin (Figure 1).

## Anaerobic digestion and nutrient recycling

Anaerobic digestion is currently often the final step of any biorefinery concept, including those involving cyanobacteria (Veerabadhran et al., 2021). As far as we know, the anaerobic digestion of cyanobacterial biomass has not yet been used at scale in industry practice. In anaerobic digestion, a consortium of different anaerobic microorganisms converts organic compounds into methane and carbon dioxide while remineralizing other elements such as nitrogen (in the form of ammonia) and phosphorous (in the form of phosphate) (Patel et al., 2021; Bohutskyi et al., 2015a, 2015b; Bohutskyi et al., 2016). Biogas, solid sludge, and liquid digestate are the main products.

The anaerobic digestion of cyanobacteria at high pH has proven a challenge. The ammonia (NH_3_) produced during digestion inhibits the growth and activity of many methanogenic microbes (Jiang et al., 2019), which are responsible for methane production during anaerobic digestion. The ammonia concentration increases exponentially with pH (pK NH_3_, NH_4_^+^ 9.2), hence, alkaline conditions are prohibitive for successful digestion of cyanobacteria (Nolla-Ardevol et al., 2015).

The remains of the biomass after phycocyanin extraction consists of intact cells of heterotrophic bacteria, cellular debris, fatty acids, hydrogen, and ammonium (Demirkaya et al., 2023; Vadlamani et al., 2021; Zorz et al., 2023). Fortunately, during the dark incubation, the pH of the medium decreases from above 10.5 to below 8, reducing ammonia toxicity. It also completely lyses the cyanobacterial cells, eliminating the need for pretreatments to lyse cyanobacteria.

Successful anaerobic digestion of dark-incubated *Ca. S. alkaliphilum* has been demonstrated (Demirkaya et al., 2025). Two lab-scale digesters were run for 600 days at ambient temperature (21°C), inoculated with sediments from the same soda lakes that were used to obtain *Ca. S. alkaliphilum*. Both digesters converted 62% of the biomass remains into biogas at a methane yield of 471 mL/g volatile solids.

This biogas can be used to drive a combined heat and power unit to produce heat and power for phycocyanin spray-drying. The concentrated carbon dioxide gas produced by the heat and power unit can be sparged into the pond to supplement direct air capture. As mentioned, the direct air capture rate may be low. However, by recycling the concentrated carbon dioxide gas produced from the bulk of the biomass, direct air capture only needs to provide the carbon that leaves the facility in the form of phycocyanin (Strous et al., 2025), usually about 20% of the biomass, and, potentially, solid digestate.

To close the overall process’s nutrient cycle, the liquid and solid digestate can, in theory, be returned to the raceway pond. However, this step has not yet been shown experimentally. Although the digestate contained sufficient nutrients for growth (Paquette et al., 2022), it was shown to also inhibit the growth of cyanobacteria (Paquette et al., 2025). Gradual adaptation of the cyanobacterial consortium to the digestate may be needed to recruit new heterotrophs able to oxidize and reuse the inhibitory substances.

## Conclusion and future directions

Here, we propose a framework for sustainable phycocyanin production by cultivating alkaliphilic cyanobacteria under high pH and high alkalinity conditions. This approach integrates four innovations: (1) direct air capture of carbon dioxide, eliminating the need for flue gas injection; (2) the use of cyanobacterial consortia rather than isolated strains to enhance culture stability; (3) passive dark incubation for phycocyanin extraction, avoiding mechanical cell lysis; and (4) recovery of energy, carbon dioxide, and nutrients from residual biomass remains via anaerobic digestion. Together, these innovations represent a path toward a more sustainable, circular bioprocess.

Within this context, several future research directions and technical challenges remain:

First, evaporative water loss remains a primary challenge in open raceway pond systems, particularly because water evaporation and carbon capture are both governed by gas–liquid mass transfer rates. In the *Ca. S. alkaliphilum* system, approximately 2,000 mol of evaporated per mol of carbon captured. Strategies that enhance mass transfer, such as more vigorous mixing or introducing nanobubbles (Ng et al., 2024) could increase the carbon capture rate but would also increase the evaporation rate. Alternatively, covering the pond with a gas-permeable but water-impermeable membrane, such as polyethylene film, could mitigate evaporation. Such materials are widely available and used in food packaging industry. Notably, polyethylene has low water permeability but remains permeable to carbon dioxide and oxygen, which is advantageous for photosynthesis.

Second, nutrient recovery from both liquid and solid fraction of anaerobic digestate was only partially successful and requires optimization. Future work could focus on developing biological pretreatments to remove inhibitory compounds (e.g., Fu et al., 2023; Zhang et al., 2023). An alternative strategy would involve engineering or selecting a cyanobacterial consortium that are tolerant to these compounds, potentially via inclusion of heterotrophic bacteria capable of effective mineralization.

Third, beyond the production of phycocyanin, the sensitivity of phycocyanin to high temperatures and low pH is currently a major barrier to market growth. Thermal degradation and pH-induced conformational changes of the phycobilin chromophore result in loss of the phycocyanin protein, limiting its application in food and breverage products that undergo heating or have low pH. To enhance the stability, stabilizing strategies, such as using additives (Chaiklahan et al., 2012; Kannaujiya and Sinha, 2016) or encapsulation techniques (Schmatz et al., 2020; Suzery et al., 2017) are under investigation. These approaches could also be applied to phycocyanin from alkaliphilic cyanobacteria.

With continued optimization, the proposed system may serve as a model for circular bioeconomy strategies, positioning cyanobacteria not only as biomass producers, but as multifunctional platforms for the production of sustainable, high-value compounds. Upon reaching industrial scale, comprehensive life cycle assessment will be essential to evaluate the system’s overall environmental and economic sustainability.

In addition to technical challenges, regulatory compliance must be addressed. Unlike *L. platensis*, alkaliphilic cyanobacteria currently lack GRAS (Generally Recognized as Safe) status, which presents a barrier to commercial deployment in food applications. Achieving such status requires extensive safety assessments and regulatory review.

At the same time, halo-alkaliphilic ecosystems, such as soda lakes, remain underexplored and harbor a wealth of untapped microbial diversity. This include both prokaryotes and eukaryotes with potentially unique metabolic adaptations that may be of broad biotechnological relevance. Systematic bioprospecting could yield novel extremozymes for industrial applications, such as alkaline proteases for detergents (Niyonzima and More, 2015), or even sources of new nanomaterials, such as diatom frustules (Hildebrand, 2008; Lopez et al., 2005). Moreover, metagenomic screening and synthetic biology may reveal heterotrophs symbionts that enhance the resilience and productivity of cyanobacterial cultures.
